# Findings of the literature review on mobility, infectious diseases and malaria

**DOI:** 10.1186/1475-2875-11-S1-P101

**Published:** 2012-10-15

**Authors:** Maxine Whittaker, Catherine Smith

**Affiliations:** 1 Asia Pacific Malaria Elimination Network (APMEN; 2 Australian Centre for International and Tropical Health, University of Queensland, Australia

## Background

Imported malaria is no longer a challenge only for malaria free countries, but for countries implementing in malaria elimination strategies and seeking to address cross-border transmission. Mobility is frequently mentioned as a risk factor and a barrier to elimination by malaria researchers and policy makers. However, only a small body of research has engaged in a detailed analysis of the links between mobility and malaria transmission, and attempts to incorporate these findings into policy are rarer still. This paper presents the findings of a literature review on malaria and human mobility supported by the Asia Pacific Malaria Elimination Network (APMEN). It attempts to shift the agenda from identifying human mobility as a risk factor, to finding strategies to work collaboratively with mobile populations towards the goal of malaria elimination.

## Materials and methods

The objectives of the literature review were to highlight key trends in the ways in which the published malaria literature discusses human mobility identify lessons to be learned from the ways that other infectious disease control programmes such as HIV/ AIDS and polio have addressed human mobility identify potential ways forward, so that it becomes possible to address human mobility within malaria elimination initiatives. The review focused upon published, peer-reviewed literature on malaria and mobility sourced through PubMed and ProQuest, and grey literature sourced though Google Scholar.

## Results

The paper will present a brief taxonomy of mobility, since there are many different form of behaviour that are often grouped together as ‘mobility’. It then discusses the three key themes that most frequently recur within the published malaria literature, namely: mobility, land use and economic change; borders; and accessing mobile populations. The paper then discusses the ways in which other infectious disease control programmes such as HIV/AIDS and polio have addressed human mobility, and to identify the key lessons to be learned from these programmes.

## Conclusions

Recommendations, methodologies, and areas that APMEN partners and other organisations may consider for future work, in order to move towards a more productive engagement with mobile populations.

